# Camel *pestivirus* infection in northern Saudi Arabia

**DOI:** 10.4102/ojvr.v93i1.2262

**Published:** 2026-06-18

**Authors:** Intisar K. Saeed, Yahia H. Ali, Muaz M. Abdellatif, Medhat A. Abu-tahon, Anwar A. Alsharari, Ahmad M. Abdel-Mageed, Husham M. Ataalfadeel, Salma Y. Alsheikh

**Affiliations:** 1 Department of Biological Sciences, College of Science, Northern Border University, Arar, Saudi Arabia; 2 National Center for the Prevention and Control of Plants Pests and Animal Diseases, Ministry of Agriculture, Arar, Saudi Arabia; 3 Department of Mathematics, College of Science, Northern Border University, Arar, Saudi Arabia

**Keywords:** *pestivirus*, camels, risk factors, serology, epidemiology, Saudi Arabia

## Abstract

*Pestivirus* infection in camels has been insufficiently studied, particularly in Saudi Arabia. In the current work, the seroprevalence of *pestivirus* infection in camels’ sera (*n* = 180) in Rafha in northern Saudi Arabia was screened using Enzyme Linked Immuno-Sorbent Assay (ELISA). Data on age, sex and breed were analysed statistically. *Pestivirus* seroprevalence was 11.1. It was higher in adults (22.2%) than in young animals (10.5%), higher in females (33.3%) than in males (10.7%) and higher in the Mjahim breed (23.8%) compared to the Magater breed (9.4%). Chi-square analysis revealed a significant correlation between *pestivirus* infection and breed (*p* = 0.015), whereas no significant associations were noted with age (*p* = 0.123) or sex (*p* = 0.432). Wald statistics indicated that breed (*p* = 0.005) and sex (*p* = 0.048) were significant predictors of *pestivirus* infection.

**Contribution:**

To our knowledge, this is the first study to report the risk factors associated with *pestivirus* infection in camels in northern Saudi Arabia. The results can help explore epidemiological parameters and aid in control, while future research should include larger samples and molecular techniques to verify active infection.

## Introduction

The dromedary camel (*Camelus dromedarius*) is a species with major agricultural importance: it is mostly maintained in desert and semi-arid regions of Northern and Eastern Africa, Asia and South America (Eckstein et al. 2022).

*Pestivirus* is known to cause infections characterised by respiratory, digestive and reproductive symptoms in cattle and other ruminants. This virus belongs to the *pestivirus* genus within the flaviviridae family (Demil et al. 2021). *Pestiviruses* are reported to cause reproductive and respiratory infections in ruminants in many countries, and the existence of the disease in camels is considered late compared to other animal species. Reports confirming Bovine Viral Diarrhoea (BVD) in different species of camelids were reviewed (Evans & Reichel 2021; Kandeel & Al-Mubarak 2022; Shah et al. 2022). Serological evidence of Bovine Viral Diarrhea Virus (BVDV) infection in camels was reported in many African countries including Sudan through the detection of its antibodies (Intisar et al. 2010) and antigens (Saeed et al. 2015), Algeria (Saidi et al. 2018), Egypt (Malek & Madkour 2017), Iran (Rasooli et al. 2023), Turkey (Nural & Sibel 2020) and Iraq (Al-Rubayie 2016).

In Saudi Arabia, work on *pestivirus* infections in camels is sparse, and Al-Afaleq et al. (2007) reported the detection of *pestivirus* antibodies. More recently, antibodies were observed in camel sera in Saudi Arabia (Al-Mubarak et al. 2022; Intisar 2019; Khalafalla et al. 2017).

This study aimed to estimate the prevalence and risk factors of *pestivirus* among *C. dromedarius* in northern Saudi Arabia, filling an epidemiological gap to assist in the disease control.

## Research methods and design

### Area of study

The study was carried out in Rafha City, located in Northern Borders province at the northeastern part of Saudi Arabia.

### Sample size calculation

The sample size was calculated according to Thrusfield (2018), using an expected prevalence of 10.8% as published by Intisar (2019), a 95% confidence interval and absolute precision of 5%. In the following equation, *p* represents the expected prevalence, *d* for the desired precision and *n* the required sample size. Substituting these values into the formula resulted in a sample size of 162. The sample size was increased to 180 to enhance precision (Equation 1):


$$n=1.96^{2}p(1-p)/d^{2}$$ [Eqn 1]

### Serum sample collection

During slaughtering, 180 blood samples were collected from clinically healthy *C. dromedarius* at the slaughterhouse. Sera were separated and used for antibody detection using ELISA.

### *Pestivirus* antibodies detection

The seroprevalence of *pestivirus* in camel sera was tested using competitive ELISA kits, purchased from Inmunología Y Genética Aplicada (INGENASA), S.A.C./Hnos. Garcia, Noblejas, 39 28037 – Madrid, Spain. The kits were used according to the manufacturer’s protocol.

### Statistical analysis

*Pestivirus* infection variables were classified, coded and saved in a Microsoft Excel worksheet, with categories for age, gender and breed. It was opened using IBM SPSS software version 27 (IBM Corp, Armonk, New York, US). The data were summarised using descriptive statistics as needed.

### Pearson Chi-square

The Pearson Chi-square test was applied to determine the association of *pestivirus* seroprevalence with age, sex and breed. Statistical significance was set at *p* < 0.05 and with a 95% confidence level.

### Binary logistic regression

Logistic regression was performed to evaluate the association between camel *pestivirus* seroprevalence with age, sex and breed.

### Model coefficients

The omnibus test was used to assess the model’s fitness, while the null hypothesis was tested using the Hosmer–Lemeshow test (Hosmer & Lemeshow 1980). Analyses were conducted at a 95% confidence level, with significance defined at *p* < 0.05.

### Ethical considerations

All ethical standards for research were followed in this study, and no direct contact with human or animal subjects was made. The study was approved by Biology Department, College of Science and Arts, Northern Border University, Arar Research Ethics (HAP-09-A-043), Kingdom of Saudi Arabia (KSA).

## Results

### *Pestivirus* seroprevalence

**FIGURE 1:**
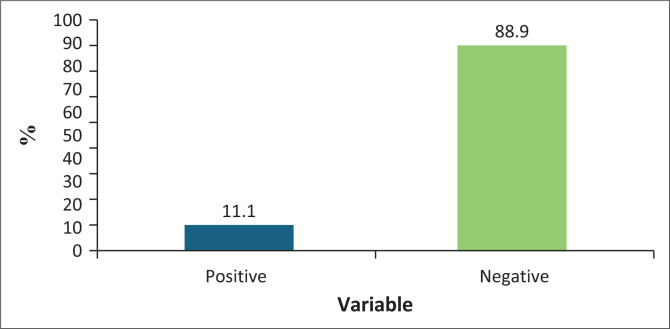
Seroprevalence of *pestivirus* in sera collected from camels as screened by ELISA.

Out of tested sera, 20 (11.1%) were reactive for *pestivirus* antibodies in camels using competitive ELISA (Figure 1).

### Seroprevalence associated with age, sex and breed

**FIGURE 2:**
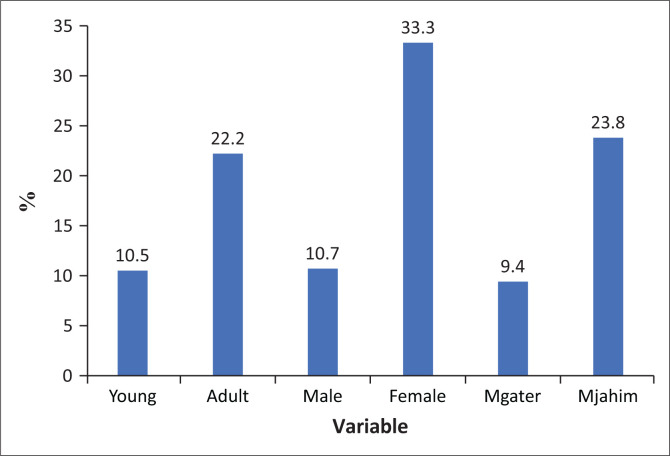
Seroprevalence of *pestivirus* in camels based on age, sex and breed as detected by competitive ELISA.

*Pestivirus* seroprevalence was found to be higher in adults (22.2%) than in young animals (10.5%), in females (33.3%) than in males (10.7%) and in the Mjahim breed (23.8%) than in the Magater breed (9.4%) (Figure 2).

### Pearson chi-square

Chi-square analysis revealed a significant association between *pestivirus* infection and breed (*p* = 0.015), whereas age (*p* = 0.123) and sex (*p* = 0.432) showed no significant association (Table 1).

**TABLE 1:** Pearson’s Chi-square analysis of *pestivirus* infection in relation to age, sex and breed.

Parameter	Age	Sex	Breed
Pearson Chi-square	2.376†	0.618†	5.859†
*df*	1.000	1.000	1.000
Asymptotic significance	0.123	0.432	0.015

*df*, degree of freedom.

† , Each table in the Chi-square test has expected a count less than five without affecting the accuracy of the test.

### Logistic regression model

Analysis revealed that the logistic regression model could be fitted as (Equation 2):


Ln(odds)=−1.792−3.036*breed+1.926*sex [Eqn 2]

Wald statistics indicated that breed (*p* = 0.005) and sex (*p* = 0.048) were significant predictors, whereas age had no significant effect. In particular, the Mjahim breed demonstrated apparent higher seroprevalence. In contrast, females had approximately 6.9-fold higher odds of *pestivirus* infection compared to males (Table 2).

**TABLE 2:** Statistical significance of variables included in the fitted mode for the prevalence of *pestivirus* infection.

Variable	B	s.e.	Wald	*df*	Sig.	Exp(B)	95% CI for Exp(B)	
							Lower	Upper
Breed	−3.036	1.078	7.939	1.000	0.005	0.048	0.006	0.397
Sex	1.926	0.975	3.900	1.000	0.048	6.864	1.015	46.443
Constant	−1.791	0.279	41.236	1.000	0.000	0.167	-	-

s.e., standard error; *df*, degree of freedom; Sig., significance; CI, confidence interval; B, beta coefficient.

## Discussion

The results of this study revealed an 11% *pestivirus* seroprevalence in camels in northern Saudi Arabia, which is very close to the prevalence previously reported in the same region (Intisar 2019).

However, it was higher than the previously reported one and the same city (Rafha) which indicates the more spread of the infection. Our detected seroprevalence was higher than the rates of 1% and 8% in other cities at northern Saudi Arabia (Al-Hammadi 2016), as well as the 4.5% recorded in eastern Saudi Arabia (Al-Mubarak et al. 2022).

Higher seroprevalence was reported in eastern and northern regions of the country (Khalafalla et al. 2017). Compared with data from other countries, our observed prevalence was higher than the 2% reported in camels in Egypt (Mahmoud & Ali 2022) and the 5% reported in Iran (Rasooli et al. 2023) and Egypt (Selim et al. 2024), as well as the 9% prevalence noted in Algeria (Saidi et al. 2018). Higher seroprevalence has been reported previously, including 14% in Iraq (Al-Rubayie 2016), 17% and 59% in Turkey (Ataseven et al. 2022; Nural & Sibel 2020), 27% and 33% in Egypt (El Bahgy, Abdelmegeed & Marawan 2018; Malek & Madkour 2017) and 85% in Sudan (Intisar et al. 2010). The variable seroprevalence could be attributed to the management system adopted as camels are mostly kept in an open system in which they can come in contact leading to the easy spread of infection.

Our results showed that a higher prevalence of *pestivirus* infection was observed among adult camels although no significant difference was observed which is most probably because of small sample size. Similar observation was reported in camels in eastern Saudi Arabia (Al-Mubarak et al. 2022) and Egypt (El Bahgy et al. 2018; Malek & Madkour 2017; Selim et al. 2024). This is expected because of the increased possibility of multiple and persistent infection with age.

Analysis by sex revealed a higher association of *pestivirus* infection in female camels than in males. In contrast, higher seroprevalence was detected in male camels in Egypt (El Bahgy et al. 2018); however, no significant association with sex was found in Saudi Arabia (Al-Mubarak et al. 2022) and Iraq (Al-Rubayie 2016). The higher prevalence detected in this study may be because of small sample size.

Regarding camel breeds, our results indicated a significant correlation between *pestivirus* infection and breed. A higher prevalence was found in Mjahim breed; however, this finding could be because of small sample size. In contrast, a weak association with breed was detected in eastern Saudi Arabia (Al-Mubarak et al. 2022). In Egypt, seroprevalence was found to be significantly higher in camels imported from Sudan compared to local breed (El Bahgy et al. 2018).

## Conclusion

This is a preliminary study; limitations were small sample size, and sampling was done in slaughterhouse rather than in farms. It was concluded from the results of this study that *Pestivirus* is circulating in camels in Rafha in Northern Saudi Arabia. Breed, sex and age were found to be correlated with pestivrus infection in camels.
